# Attitudes of legal guardians and legally supervised persons with and without previous research experience towards participation in research projects: A quantitative cross-sectional study

**DOI:** 10.1371/journal.pone.0256689

**Published:** 2021-09-15

**Authors:** Cedric Brune, Ulrike Stentzel, Wolfgang Hoffmann, Neeltje van den Berg

**Affiliations:** Institute for Community Medicine, University Medicine Greifswald, Greifswald, Germany; Imam Abdulrahman Bin Faisal University, SAUDI ARABIA

## Abstract

**Background:**

Vulnerable groups, e.g. persons with mental illness, neurological deficits or dementia, are often excluded as participants from research projects because obtaining informed consent can be difficult and tedious. This may have the consequence that vulnerable groups benefit less from medical progress. Vulnerable persons are often supported by a legal guardian in one or more demands of their daily life. We examined the attitudes of legal guardians and legally supervised persons towards medical research and the conditions and motivations to participate in studies.

**Methods:**

We conducted a cross-sectional study with standardized surveys of legal guardians and legally supervised persons. Two separate questionnaires were developed for the legal guardians and the supervised persons to asses previous experiences with research projects and the reasons for participation or non-participation. The legal guardians were recruited through various guardianship organizations. The supervised persons were recruited through their legal guardian and from a previous study among psychiatric patients. The data were analysed descriptively.

**Results:**

Alltogether, 82 legal guardians and 20 legally supervised persons could be recruited. Thereof 13 legal guardians (15.6%) and 13 legally supervised persons (65.0%) had previous experience with research projects. The majority of the guardians with experience in research projects had consented the participation of their supervised persons (n = 12 guardians, 60.0%; in total n = 16 approvals). The possible burden on the participating person was given as the most frequent reason not to participate both by the guardians (n = 44, 54.4%) and by the supervised persons (n = 3, 30.0%). The most frequent motivation to provide consent to participate in a research study was the desire to help other patients by gaining new scientific knowledge (guardians: n = 125, 78.1%; supervised persons: n = 10, 66.6%).

**Conclusions:**

Overall, an open attitude towards medical research can be observed both among legal guardians and supervised persons. Perceived risks and no sense recognized in the study are reasons for not participating in medical research projects.

## Introduction

Vulnerable persons are often excluded from research projects. One consequence is that little research is done to solve specific problems in the medical care for these people, although there is a great need [1–3]. It is important to recruit a sufficient number of vulnerable persons for studies to obtain representative results for these groups to let them participate in medical progress [1]. A better understanding of the decision-making process of vulnerable persons and their guardians can help to create a better framework for research with vulnerable patients [4] which increases the chance of generating higher numbers of participants in a legally secure manner [5].

The group of vulnerable persons includes people with psychiatric disorders, with dementia and with neurological disorders [6]. One problem is that these persons are often unable, either completely or in specific domains (e.g. in financial or health matters), to take care of their affairs independently. In many cases, these people are supported by legal guardians [7]. In Germany, legal guardians are appointed by the court. The court also determines the tasks of the legal guardians. Legal guardians can support in healthcare matters, financial management, housing and administrative matters, and any of the combinations of these domains [8].

In 2014, around 6% of the German population was over 80 years old. By 2050 their share will rise to nearly 13% [9]. In Germany, the prevalence of dementia in 65-69-year-olds is 1.3%, rising to 22.8% for 85-89-year-old people [10]. There are currently about 1.7 million people living with dementia in Germany. By 2050, the number of people affected is expected to double [11]. Worldwide the number of people living with dementia was about 35 million in 2010. The number is projected to reach over 65 million in 2030 [12]. Associated with the demographic ageing of many societies the number of people with dementia in need of care by a legal guardian will rise [7].

In the younger age groups, severe mental illness is often a reason why support by a legal guardian is needed. The lifetime prevalence of mental illness is about 26% [13, 14]. The prevalence of severe mental illness in the total population of Germany is estimated at 1–2% [15].

In total, about 1.3 Mio. people in Germany have a legal guardian (2015) [16]. People with dementia represent the majority of all court-ordered guardianship (34%). In a German study in nursing homes, the proportion of people with dementia who have a legal guardian was 39% [5]. This proportion is 13% among persons with psychoses or stress and personality disorders, who represent the second largest group [7].

Women are more likely than men to receive support from a legal guardian. However, there are considerable variations between the different age groups. In the age group 45-65-years, men (55%) dominate, whereas among the over 90-year-olds, women (72%) represent the larger share of those who are cared for. This can be explained by the higher life expectancy for women in Germany [7].

The Helsinki Declaration deals with the ethical principles for medical research in humans. One aspect of the declaration is that underrepresented groups should have adequate access to research and to the results of research [17]. However, better care for vulnerable patient groups can only be achieved if they are not systematically excluded from clinical trials and studies in health services research. By gaining new knowledge, a reduction of disadvantages towards these groups of people is likely in the future [3]. Sutton et al. (2003) states that research should be equally accessible to everyone, regardless of age, social status, or disease [1]. Dunn et al. (2011) support a similar view. Dunn et al. call for more research on the decision-making process of legal guardians with respect to the participation of legally supervised persons in research projects [4].

This study aims to identify motivations for the decision of both legal guardians and legally supervised persons for participation in research projects. With a better understanding of who is involved in the decision-making process and what aspects influence decision-making, study protocols can be better adapted to the needs of vulnerable groups and their legal guardians. In addition, the consent can be adapted to new findings, whereby an increase of study participation of vulnerable people can be achieved.

## Methods

### Recruitment of legal guardians

**Fig 1:**
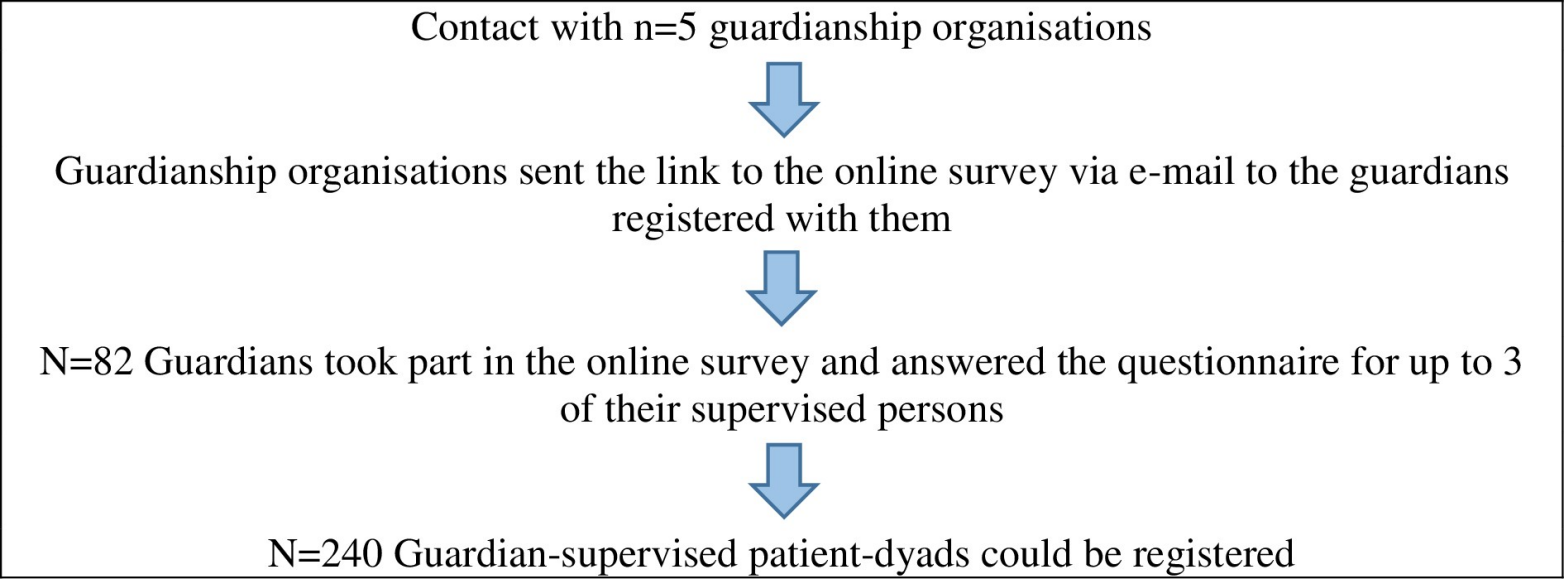
Sampling of guardian-supervised patient-dyads.

A legal guardian (also called “supervisor”) is ordered for adult persons who are unable to manage their affairs independently due to mental, physical, or intellectual disability, to manage defined tasks of these persons or support them with these tasks (Section 1896 Paragraph 1 German Civil Code). The legal guardians were recruited through various guardianship organizations. The federal association for professional legal guardians (Bundesverband für Berufsbetreuer e.V.) and the healthcare authorities of the districts of Vorpommern-Greifswald, Vorpommern-Rügen, Rostock, and Schwerin sent the link to the online survey via e-mail to the guardians registered with them. The legal guardians were asked to answer questions for up to three of their supervised persons (Fig 1). The inclusion criterion was that all participants had to be employed as a legal guardian. We do not have exact information about the number of legal guardians who received the questionnaires, because the distribution was done through their guardianship-organisations. In the consequence, we can not make detailed information about the response rate.

### Recruitment of the legally supervised persons

Legally supervised persons were recruited 1. by contacting participants of a previous study concerning patients with schizophrenia and bipolar disorders [18] and 2. by asking the legal guardians to sent the link with the survey to eligible supervised persons. The participants gained from the previous study were interviewed through telephone interviews. The responses of the persons recruited by their guardians were received online, by mail or by personal interview. In the letters to the guardians, we asked them to forward the questionnaires to their supervised persons. In the consequence, we do not have exact knowledge about the number of legally supervised persons who have received the questionnaires. Inclusion criterion was that there had to be legal care for the participant.

### Questionnaires

Two separate questionnaires were developed to assess the motives for the decision for or against participation in a research project of the guardians and the legally supervised persons, respectively. After comprehensive research in the literature, we didn’t find suitable existing questionnaires eligible to answer our research questions. Consequently, we developed our own questionnaire, tailored to the research question. Therefore, they could not be validated beforehand. The questionnaires were pre-tested by 6 legal guardians, 3 supervised persons and 2 persons who were familiar with the work of legal supervisors.

The legally supervised persons were asked for their attitude towards medical research in general, whether they have ever been asked to participate, and for reasons and motivations to participate or not in research projects.

The guardians were asked to fill in analogue questions for up to three of their supvervised persons. The second part of the questionnaire of the legal guardians aimed to assess the personal attitude of the guardians towards medical research, for which kind of supervised persons (e.g. persons with dementia, persons with mental disorders, persons with limited intelligence) he/she would approve research participation and who in the „guardian-supervised patient-dyads”makes the decision for or against participation.

The design of the questionnaires, the data assessment, and the storage of the data was conducted using the web-based survey software EvaSys©. The data collection was carried out between 06/13/2019 and 01/01/2020.

### Data analysis

The data was analyzed descriptively. Factors associated with participation were examined in multivariate models. The statistical analysis was conducted using the statistical software SPSS Statistics Version 25.0 (IBM Corp., Armonk, New York). Charts and tables were created using MS Excel 2016 (Microsoft Corp., Redmond, Washington).

### Ethics

Possible participants of the survey were informed about the need for the study, background information, and the objective of the study. In addition, data protection information was provided. The consent of the persons participating in the online survey was confirmed by clicking on a respective button. Those who participated in the study via telephone interview were given this information in an explanatory conversation at the beginning of the telephone call. All data were collected anonymously, so that no conclusions could be made about a specific person. All data were used only for the purpose of this study. In case of questions, it was possible to contact the authors via e-mail.

The Ethics Committee of the University Medicine Greifswald approved this study (registration number BB 073/19).

## Results

### Characteristics of the legal guardians

In total, 82 legal guardians participated in the survey, thereof 39 (47.6%) were male. Most of the responding guardians were between 51 and 60 years old (40.2%, n = 33).

The majority of the legal guardians had a university degree (64.6%, n = 53), followed by a university entrance qualification (18.3%, n = 15) (Table 1).

**Table 1:** Characteristics of legal guardians (n = 82).

		n	percent
**Age group**			
	< 30	2	2.4
	30–40	10	12.2
	41–50	21	25.6
	51–60	33	40.2
	> 60	16	19.5
**Sex**			
	Female	39	47.6
	Male	43	52.4
**Educational level**			
	Secondary school degree (9 years)	2	2.4
	Intermediate school certificate (10 years)	9	11.0
	University entrance qualification (12 years)	15	18.3
	University degree	53	64.6
	PhD	3	3.7
**Type of guardianship**			
	Professional	78	95.1
	Honorary	4	4.9

The legal guardians were asked to answer questions with respect to up to three of their supervised persons. In total, the 82 legal guardians answered questions to 240 supervised persons. Dementia (n = 43, 17.9%), mental illness (except schizophrenia) (n = 42, 17.5%), and schizophrenia (n = 41, 17.0%) were the most frequent reasons for court-ordered guardianship for the assessed guardian-supervised patient-dyads. Intellectual disability (n = 39, 16.2%), low intelligence (n = 24, 10.0%), neurological deficits (n = 20, 8.4%), and neglect (n = 9, 3.8%) were further reasons for court-ordered supervision. Other reasons for supervision were found in 9.2% (n = 22) of the assessed guardian-supervised patient-dyads.

### Characteristics of the legally supervised persons

N = 20 legally supervised persons were recruited to take part in the survey. Of these, 8 persons (40.0%) were recruited by their legal guardian. A further 12 persons (60.0%) could be recruited from the previous study of the Institute for Community Medicine [19].

N = 11 of the supervised persons (55.0%) were male. 45.0% (n = 9) of the respondents were between 30 and 40 years old. Younger than 30 years were 10% (n = 2).

N = 6 of the respondents answered that they manage their household alone (30.0%), 8 respondents manage the household together with other persons (40.0%). N = 3 persons each lead the household with the support of a nursing service (15.0%) or live in a nursing home (15%) (Table 2).

**Table 2:** Characteristics of legally supervised persons (n = 20).

		n	percent
**Age group**			
	< 30	2	10.0
	30–40	9	45.0
	41–50	3	15.0
	51–60	3	15.0
	61–70	3	15.0
**Sex**			
	Female	11	55.0
	Male	9	45.0
**Living situation/household**			
	Alone	6	30.0
	With others	8	40.0
	With support of a nursing service	3	15.0
	Nursing home	3	15.0
**Prior contact with research**			
	Yes	13	65.0
	No	7	35.0

### Attitudes of legal guardians towards medical research

An open attitude towards medical research was reported by 72.0% (n = 59) of the legal guardians. N = 19 (23.2%) described their attitude towards medical research projects as neutral. N = 3 (3.7%) of the guardians had no prior exposure to medical research.

N = 20 (8.3%) of the supervising-dyads had experienced at least one request to participate in a research project, this affected n = 13 guardians. The majority of the guardians with a previous research contact stated that their supervised person had agreed to participate (80.0%, n = 16).

**Fig 2:**
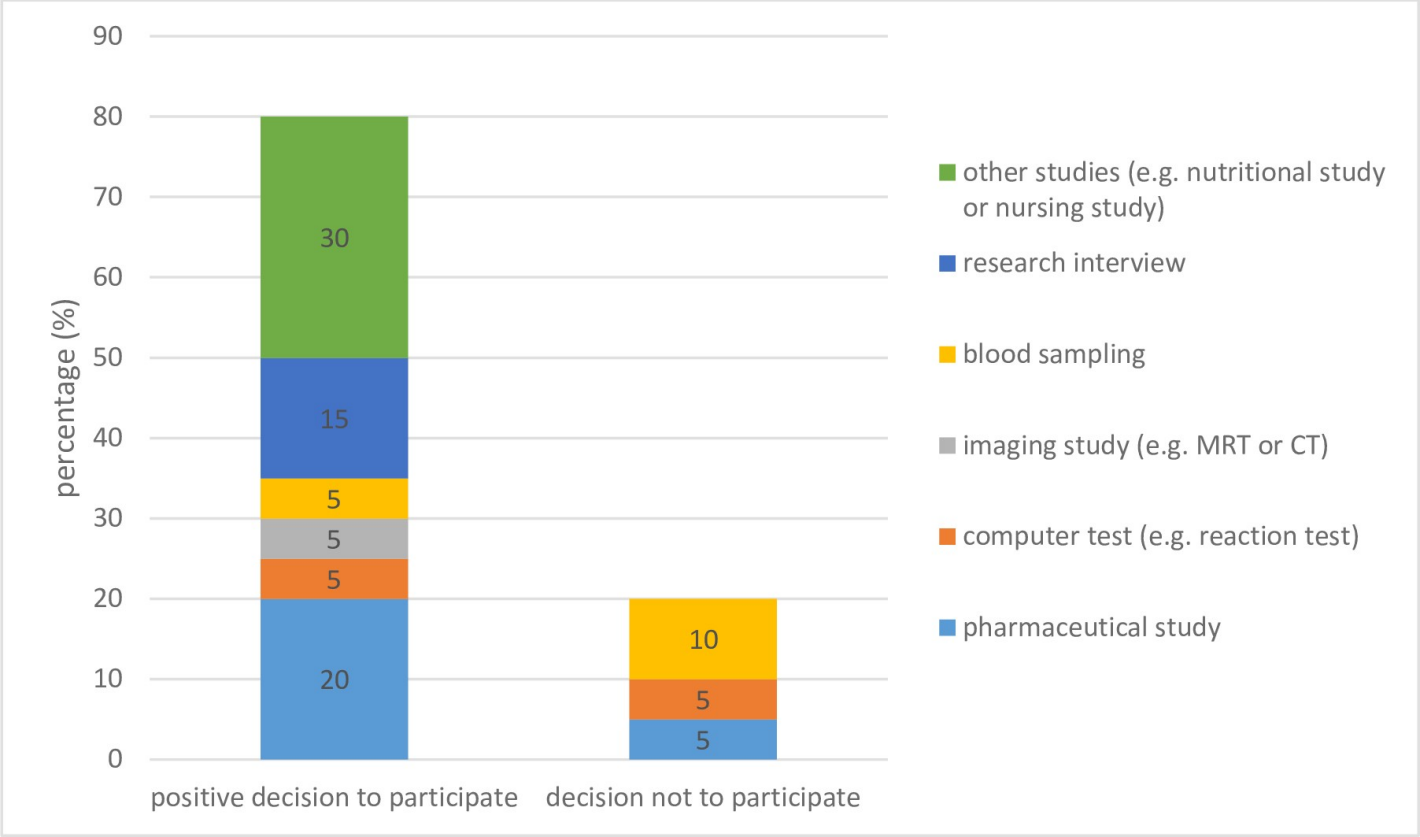
Kinds of studies where supervised persons were asked to participate (answered by the legal guardians).

N = 5 (25.0%) of the guardians with prior contact to research had been asked whether their supervised person would participate in a pharmaceutical study, 15.0% (n = 3) in an interview study. N = 3 (15%) were asked to provide blood samples for research purposes. N = 2 guardians (10,0%) were asked to give permission to participate in an imaging study (MRT or CT) (Fig 2).

In the cases of dissent (n = 4), there was one request to participate in a pharmaceutical study, one to participate in a computer-based digital questionnaire and twice to participate in studies that required blood samples to be taken (Fig 2).

### Attitudes of legally supervised persons towards medical research

N = 13 of 20 supervised persons (65.0%) had already been asked to participate in a research project.

One supervised person had been asked to participate in a computer-based digital questionnaire. N = 12 persons (92.0%) had been asked to participate in a study for persons with mental health disorders with a telemedical intervention (these were the persons, who were recuited from the previous study in the Institute for Community Medicine). Of these one person also had been asked to participate in a study in which blood was sampled for research purposes.

All supervised persons who were asked to participate in a research project had agreed (100%, n = 13). The decision was made independently by 9 of the supervised persons (45.0%). N = 2 (15.0%) made the decision together with their guardian. One supervised person stated that the decision was made by the guardian alone. One person consulted family members for advice in the decision.

N = 12 of all respondents (60.0%) could basically imagine participating in a medical research project. N = 7 (35.0%) would reject this. One person did not answer the respective question. Of those who had participated in a research project in the past (n = 13), 8 (61.5%) would consider to participate again. N = 4 persons (30.8%) stated that they do not want to participate in a research project again.

### Decision-making process in case of a request to participate in a medical research project

#### Perspective of legal guardians with prior experience with requests for study participation

N = 12 (60.0%) of the guardians stated that they had made the decision to participate for their supervised person themselves without involving the supervised person. In 1 of 20 cases (5.0%), the supervised person made the decision by him/herself. In 6 cases (30.0%), the guardian made the decision together with the supervised person.

The guardians who stated that they had made the decision by themselves (n = 12) were asked whether the supervised person agreed with this decision. In three cases (31.0%), the guardian reported that the supervised person agreed with the decision. In two cases (15.0%), the supervised person did not agree with the decision. N = 7 of these guardians (54.0%) could not report whether or not the decision was made in agreement with the supervised person.

#### Perspective of legal guardians without prior experience in requests for study participation

In the supervising-dyads in which there had been no prior request for particpation in a medical research project (n = 220), the guardians were asked, whether they would, in principle, support that one or more of their supervised persons participates in a research project. In 142 of the supervising-dyads (64.5%), the guardian stated that he would agree.

With regard to the question of who would probably make the decision to participate or not, n = 195 dyads could be analyzed. In 12.8% (n = 25) of these, the guardian would make this decision by him/herself. In 36.0% (n = 9) of these cases the guardian-supervised patient-dyad exists because of a dementia and in 28.0% (n = 7) because of an intellectual disability. In 29.2% of these dyads (n = 57), the guardian would leave the decision to the supervised persons. In these cases the most frequent reason for guardianship is mental illness (except schizophrenia) (n = 17, 29.8%), or schizophrenia (n = 16, 28.1%). In 53.3% of the dyads (n = 104), the guardian would make the decision jointly together with the supervised person. In this subgroup the most frequent reason for care is a intellectual disability (n = 20, 19.2%). In 4.6% of the dyads (n = 9) the guardian would involve family members of the supervised person in the decision.

### Reasons and motivations for agreeing to participate

#### Perspective of the legal guardians

N = 160 dyads would agree in principle with the particpation of supervised persons in a medical research project.

**Fig 3:**
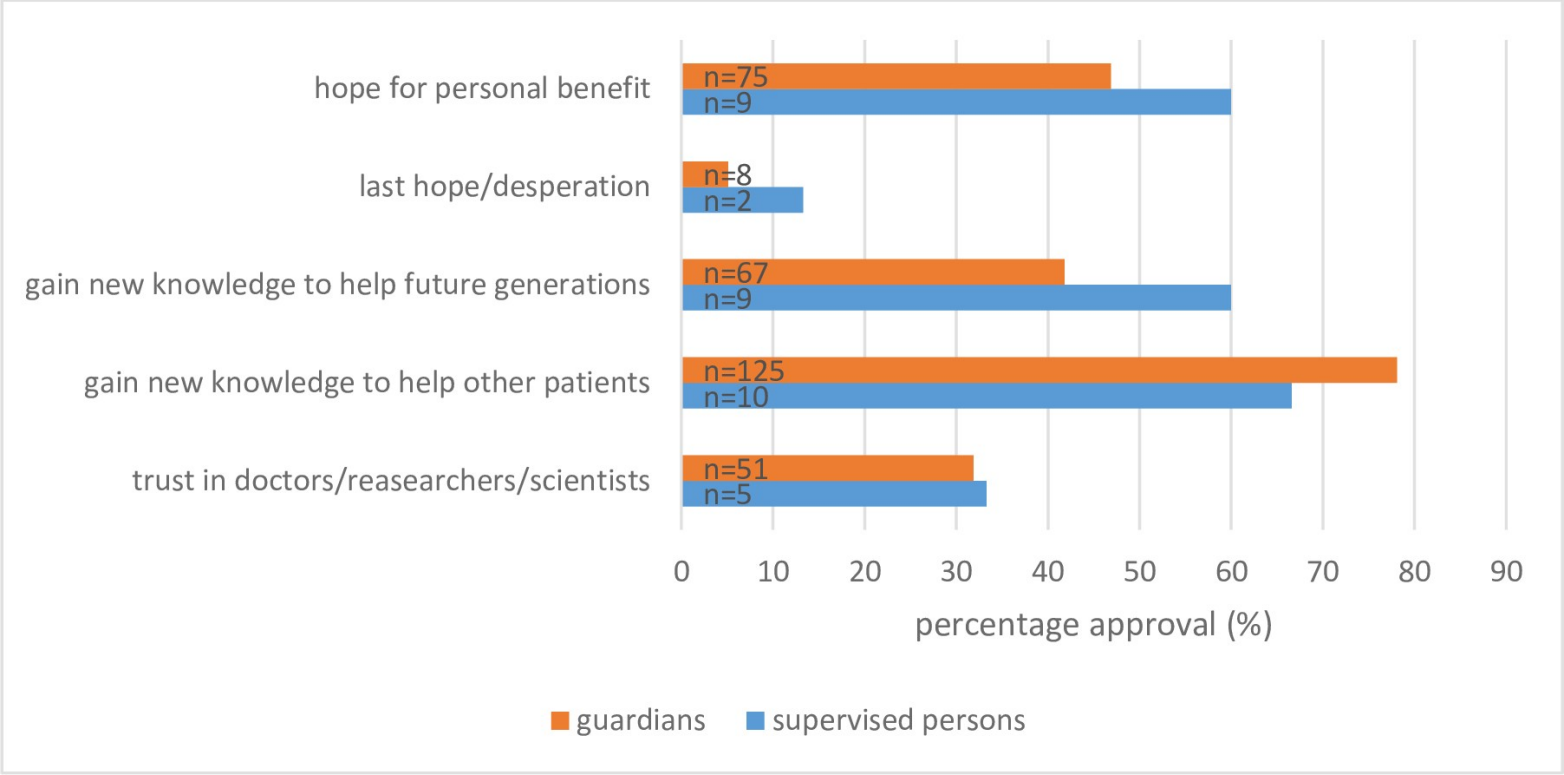
Reasons/motivations for agreeing to participate in a medical research project from the perspective of the guardians for each guardian-supervised patient-dyad separate (n = 160) and the supervised persons (n = 15). Multiple answers were possible.

The most frequent motivation for agreeing to participate was to help other patients (78.1%, n = 125). For 41.8% (n = 67) the gain of new knowledge to help future generations was one reason to participate. For 46.9% (n = 75), a personal benefit for the supervised person would be a motive for agreement. 31.9% (n = 51) expressed confidence in doctors and scientists as one of the possible reasons for consent (Fig 3).

#### Perspective of the supervised persons

N = 15 supervised persons with no prior exposure to a research study responded to the question, whether they would participate in research projects. Thereof, n = 12 persons could imagine to participate in a research project. N = 9 persons (60.0%) mentioned the hope of personal benefit as a reason for consent. Other reasons for participation were altruistic motives. These included participation with the hope of gaining new knowledge to help future generations (60.0%, n = 9) and gaining new knowledge to help other suffering people (66.6%, n = 10). N = 5 respondents (33.3%) see their approval as being based on their trust in scientists, researchers, and doctors. N = 2 supervised people (13.3%) see participation in medical research projects as a last hope for cure. One person mentioned a contribution to the general progress of research as a motivating factor. One person wanted a better understanding of their own illness and one person mentioned financial compensation as a possible reason for participation (6.8%) (Fig 3).

### Reasons for not participating in a medical research project

#### Perspective of the legal guardians

For n = 80 of the guardian-supervised patient-dyads, the guardians would rather not agree to participate in a medical research project.

**Fig 4:**
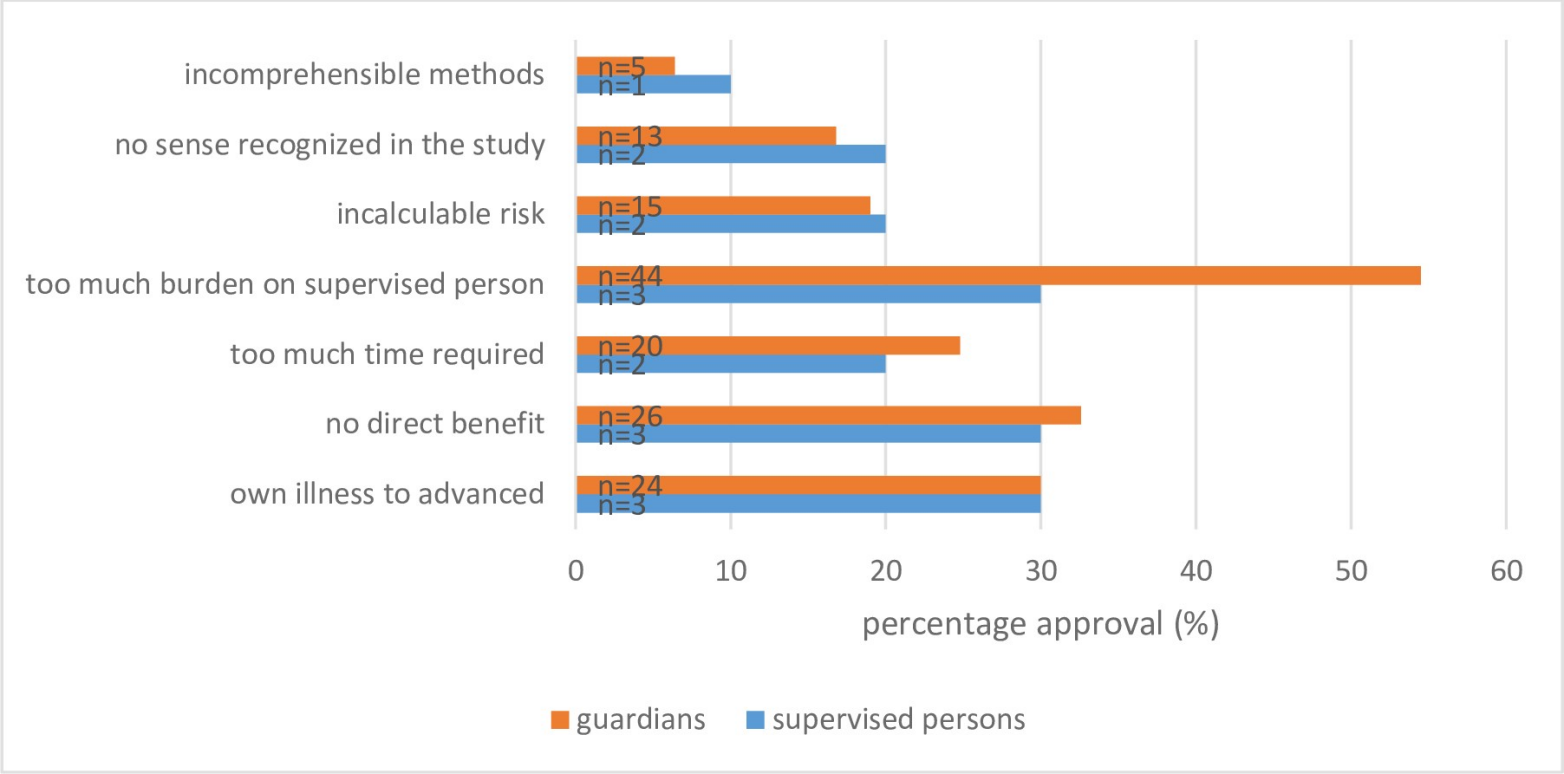
Reasons for disagreeing participation in a medical research project from the perspective of the guardians for each guardian-supervised patient-dyad separated (n = 80) and supervised persons (n = 10). Multiple answers were possible.

In 54.5% (n = 44) of these dyads, the reason was the anticipated burden on the supervised person. No personal benefit for the supervised person would be a reason for not agreeing to participation in another n = 26 cases (32.6%). For n = 20 dyads, the required time for participating in a research project would be a reason for not agreeing (24.8%). For n = 15 dyads (19.0%), an incalculable risk for the supervised person would be a reason for the guardian not to agree participation (Fig 4).

#### Perspective of the legally supervised persons

N = 10 legally supervised persons answered the question soliciting motivations not to particpate in a medical research project.

Frequent reasons include the burden on themselves (30%, n = 3), the advanced stage of their disease (30%, n = 3). or not having a direct benefit (n = 3, 30%). N = 2 supervised persons (20%) mentioned an incalculable risk as reason for non participation. Another 2 supervised persons (20%) mentioned a general scepticism against research as a reason for non participation. One supervised person did not have an understanding of what research means (Fig 4).

## Discussion

We analyzed the reasons for legal guardians and legally supervised persons for ageeing or not agreeing to the participation of patients under legal guardianship in medical research projects.

N = 20 (8.3%) of the guardian-supervised patient-dyads (n = 240, total), had experienced a request to participate in a medical research project. On the side of the legally supervised persons, the proportion was 65.0% (n = 13). The fact that 12 patients in this group were recruited from prior projects has likely biased this high proportion.

For the guardians, the most frequent request had been to agree to the participation of their supervised persons in pharmaceutical studies (20%, n = 4). They had also experienced requests to the participate in studies that included blood sampling for research purposes (15%, n = 3), studies that included research interviews (15%, n = 3), and imaging (10%, n = 2). The guardians consented in 16 of the 20 cases (80%). The numbers are too small, to interprete an association between the kind of study and the willingness to approve a participation.

In the literature, in most studies the decision on participation in research studies for vulnerable people was investigated for people with Alzheimer’s disease [4, 19–22] or mental illness [23]. The decision-making process was often studied for cases where a family member was the guardian [19, 24].

The approval rate for participation in research projects with Alzheimer’s patients decreases with an increasing risk profile of the supervised persons, probably because guardians more often see the risk rather than the benefit of new treatment concepts [4, 21]. Compared to the study by Dunn et al., our study does not only examine the willingness to participate in medical research in Alzheimer‘s patients but also in other vulnerable groups. Therefore, our results are not fully transferable to studies with Alzheimer‘s patients.

According to our study, altruistic motives, such as helping others suffering from the same disease and helping future generations, are the main reasons for the participation in research projects for both the legal guardians and the legally supervised persons. Dunn et al. also identified altruistic motives as reasons for approval. According to Dunn et al., it is not always clear whether the guardian indicates altruistic motives in order to be able to benefit from the participation of his supervised people in the future [4] Just as with Black et al., in our study, supervised persons gave as a reason for their willingness to consent that they want to help people in the future. A further reason for the consent that dementia patients mentioned in the study by Black et al. was that the guardians could then understand the situation of the supervised person better [25].

Trust in scientists, researchers, and doctors was also mentioned as a reason for research approval. Here, the views of the legal guardians and the legally supervised persons coincide. Similar views are expressed in a study by Sugarman et al. (2001). However, in this study only persons who gave their consent to participate in research projects were asked [21]. According to Bond Sutton et al., distrust in research is one reason why guardians do not give consent to research projects for their supervised persons. Another reason for rejection is the fear that proven treatment concepts will no longer be unconditionally accessible under the protocol of a clinical study [1]. Few legal guardians see their supervised person in such a situation that they would give consent on the grounds of last hope or despair.

From the perspective of the guardians one important reason for not agreeing to participation in research projects is that a medically unnecessary measure would be too much of a burden for the supervised person, especially if no direct benefit is expected for the participant. Both the studies of Sugarman et al. [21] and Black et al. [25] confirm our results. In some cases, guardians indicated that they did not want to give their consent because the illness of the supervised person was too severe. Roberts and Kim also found that burden and safety aspects are particularly important for people who are ill [23]. Overton et al. identified three main reasons that influence the decision for or again a consentfor participation: verbal or non-verbal indications that the guardians can identify in the person he is supervising, sickness and care experiences, and fundamental values of the supervised person and the guardian [26].

The reasons for approval or refusal to participation in research projects must be interpreted with caution, as it was not possible to simulate a real situation. Roberts and Kim (2015) also caution that decisions regarding participation in research projects have more theoretical significance due to their rarity [23]. Shalowitz et al. (2006) note the possibility of differences between answers to hypothetical decisions and to decisions that would affect real life [27].

Half of the supervised persons included in our study stated that they would like to decide for themselves about participation or non-participation in a medical research project. 25% would prefer to make the decision together with their guardian. Few of the supervised persons indicated that they would include family members in the decision. Only one person under supervision stated that he or she would let the decision be made by the guardian alone. Unlike our finding Kim et al. (2009; 2013) reported that a large proportion of people under supervision is willing to delegate some or all of their decision-making competencies to guardians [20, 28]. The results of the survey of the legal guardians showed consistent results compared to those of the supervised persons. Black et al. (2013) found that the more cognitively impaired the person receiving supervision is, the more often discrepancies with the view of the guardian occur [25]. Discrepancies can also be explained by the fact that the guardian and the supervised person rarely exchange information on research-related issues [4]. Our results contradict those of Black et al., where the majority of caregivers stated that they would make their own decisions on future research decisions, or would involve family members [25].

Concerning the question whether guardians would disregard the presumed will of the supervised persons, some guardians indicated that they would do so if an advantage for the person they were supervising could be expected from participating in a research project. This answer is most compatible with the best-interest approach to decision making [19, 26, 29]. The best-interest approach is often contrasted with substituted decision making, which should reflect the presumed will of the supervised person [29]. A decision is therefore always difficult to make, as the will of the cared person must be respected, but also the person’s well-being has taken into account. Nevertheless, disregarding the presumed will of the supervised person raises fundamental ethical questions. In the case of the involvement of legally supervised persons in medical research, we believe that it is essential to inform the legal representatives about applicable guidelines and ethical standards. Since no significant influences on attitudes toward medical research could be identified, we can’t draw conclusions on possible age-, gender- or education-specific strategies to inform guardians about research projects.

## Limitations and strengths

### Limitations

The samples of the guardians and supervised persons are not representative for all guardians and all supervised persons in Germany. Many of the supervised persons have severe cognitive limitations or a diminished intelligence and are not able to fill out written questionnaires. Therefore, the guardians didn’t forward the questionnaires to their supervised persons in most cases. To include at least a small sample of supervised persons, we also included participants of a previous study, which are of course not representative because they all had experience in participating in a research project.

The low sample size of supervised persons is a limitation of our study. It is important to keep in mind that it is extremely difficult to recruit supervised persons for research projects in general. Care organisations do not provide direct information about supervised persons. So the only option is to try to contact them via their legal guardians. In addition, these persons often have a limited cognition or intelligence and do not react by themselves on such requests.

In our design the legal guardians selected up to 3 of their supervised persons. While this approach his broadens the sampling frame, the choice of the legal guardians may have been biased limiting the representativeness of the guardian-supervised patient-dyads assessed in this study.

### Strengths

To our knowledge this is the first study in Germany assessing reasons for and against participation of supervised patients in medical research. With a sample of 80 participating caregivers, a large sample was examined. Our study not only examined attitudes towards research in Alzheimer’s patients or patients with mental illness but also included patients with an array of reasons for court-ordered supervision. Moreover, in most of the internnational studies, the legal representatives were mostly family members. Our study includes both guardians and supervised persons, who had already been exposed to medical research as well as those without previous experience.

## Conclusion

Our study could show that there is generally a positive attitude towards medical research among legal guardians and supervised persons and both regard participation in medical research projects predominantly positive. Perceived risks and no sense recognized in the study are possible reasons for rejection of medical research projects. A complete and comprehensible explanation of the project by the scientists and researchers to the legal guardians is essential. In order to obtain the consent of the supervised person, the explanation of the study protocol should be given in simple language, because supervised persons may have a difficult understanding of complex issues. The information should mention not only the risks but also the potential benefits, as this enables guardians and supervised patients to weigh the risks and benefits adequately.

Because of demographic change and the associated increase in the number of people in need of supervision and assistance [7], it is important to improve access to these vulnerable groups of people who are underrepresented in research.

## Supporting information

S1 File Questionnaire legal guardians German. (PDF)

S2 File Questionnaire legal guardians English. (PDF)

S3 File Questionnaire legally supervised persons German. (PDF)

S4 File Questionnaire legally supervised persons English. (PDF)

S5 File Data set legal guardians. (CSV)

S6 File Data set legally supervised persons. (CSV)

S7 File (CSV)

S8 File (CSV)
