# Supplementary material for: Attitudes of legal guardians and legally supervised persons with and without previous research experience towards participation in research projects: A quantitative cross-sectional study
Source: PLoS One. 2021 Sep 15;16(9):e0256689. doi: 10.1371/journal.pone.0256689 (PMC8443074; doi:10.1371/journal.pone.0256689)
Supplement: S1 File — (PDF) [file pone.0256689.s001.pdf]

MUSTER

EvaSys	Questionnaire legal guardians	Electric Paper

Universitätsmedizin Greifswald

Institute for Community Medicine, Department Guardianship study 2019

Bitte so markieren: ☐ ☒ ☐ ☐ ☐ Bitte verwenden Sie einen Kugelschreiber oder nicht zu starken Filzstift. Dieser Fragebogen wird maschinell erfasst.

Korrektur: ☐ ■ ☐ ☒ ☐ Bitte beachten Sie im Interesse einer optimalen Datenerfassung die links gegebenen Hinweise beim Ausfüllen.

1. Declaration of consent

Dear legal guardian,

Thank you for taking the time to answer the following questionnaire.

With your help, we, scientists of the University Medicine Greifswald, would like to examine your motives for or against participation in medical research projects.

In the future, it will become more important that legally supervised persons can also participate in medical research projects. In this questionnaire, you will first be asked some questions about your guardianship. Then we would like to know more about your motives for or against participation in research projects.

There is no "right" or "wrong" in answering the questions; only your opinion matters. Answering the questionnaire will take about 10 minutes.

We will treat your data with strict confidentiality. All data are subject to data protection and will only be processed anonymously and will not be passed on to third parties. Your data will be used exclusively for the scientific purpose of this study and will not be used for any other purpose afterward. Participation in the survey is voluntary.

In principle, the decisions of a legal guardian are to be made in such a way that the best interests of the legally supervised persons are served and that the person is not patronized. Important matters are to be decided in consultation with the legally supervised person, provided that this does not result in any disadvantage for the supervised person [§1901 Abs. 3 BGB].

If you have any questions about this survey, please feel free to contact us via e-mail (betreuerstudie@uni-greifswald.de).

1.1

☐ I have taken note of the text above and agree to participate in this study.

2. Sociodemographic questions

2.1 Please state your age (in years)

☐ under 30 ☐ 30-40 ☐ 41-50
☐ 51-60 ☐ over 60

2.2 Please state your gender

☐ Female ☐ Male ☐ Diverse

2.3 What is your family status?

☐ Unmarried ☐ Married ☐ Living permanently apart
☐ Divorced ☐ Widowed

2.4 What is your highest education level?

☐ No graduation ☐ Secondary school degree (9 years) ☐ Intermediate school certificate
☐ University entrance qualification (12 years) ☐ University degree ☐ PhD
☐ Habilitation

2.5 What are you by profession?

[ ]

2.6 What type of guardianship do you provide?

☐ honorary ☐ professional

2.7 How many persons do you currently supervise?

☐ 1 ☐ 2 ☐ 3
☐ 4-6 ☐ 7-9 ☐ 10-12
☐ 13-15 ☐ 16-18 ☐ 19-21
☐ more than 21

2.8 What is your attitude toward medical research?

☐ Positive attitude ☐ Neutral attitude ☐ Negative attitude
☐ Not thought about it yet

MUSTER

2. Sociodemographic questions [Fortsetzung]

2.9 What kind of study could you imagine participating in?
Multiple choice possible

- ☐ Computer test procedures (e.g. test to determine IQ or reaction time)
- ☐ Imaging studies (e.g., MRI examination to visualize altered brain structures in dementia patients)
- ☐ Study of genetic markers (important in the context of diseases with a hereditary component)
- ☐ Research interview
- ☐ Telemedical study (e.g. conducting doctor-patient conversations via video call or contact via SMS)
- ☐ Blood sampling for medical research purposes
- ☐ Others
- ☐ No participation

2.10

MUSTER

3. Note

3.1 Since you care for more than one person, please select 2 or 3 of the people you supervise. Please answer the following questions for these persons one after the other. You will be informed by corresponding notes at which point the questions for the 2nd or 3rd person should be answered.

If you have been asked to participate in a medical research project for one of your supervised persons in the past, please answer the questions related to this person.

If you have not been asked to participate in the past, please select 2 or 3 of the persons you supervise with as different reasons for supervision as possible.

☐ OK

4. Note

4.1 All the following questions please refer to the 1st person you have chosen.
☐ OK

5. Specific questions about research I

5.1 What is the reason for the supervision?
☐ Intellectual disability ☐ Dementia ☐ Schizophrenia
☐ Mental illness except schizophrenia ☐ Neurological deficits ☐ Neglect
☐ Low intelligence ☐ Others

5.2
[ ]

5.3 What domains are under supervision?
Multiple choice possible
☐ Financial management ☐ Healthcare matters ☐ Residential matters
☐ Housing matters ☐ Administrative matters ☐ Others

5.4
[ ]

5.5 What is the age of the person you are supervising?

(In case of a request for study participation for this person, please indicate the age at the time of the request).
☐ under 30 ☐ 30-40 ☐ 31-40
☐ 41-50 ☐ 51-60 ☐ 61-70
☐ 71-80 ☐ 81-90 ☐ over 90

5.6 Since when you supervise this person? (in years)
☐ under 1 year ☐ 1-3 ☐ 4-6
☐ 7-9 ☐ 10 or more years

5.7 How close is the relationship with this person?
☐ Emotional ☐ Friendly ☐ Factual
☐ No accurate assessment

5.8 Should you made a decision in the past for this person to participate or not in research project?
☐ Yes ☐ No

5.9 What kind of study was it?
☐ Pharmaceutical study ☐ Computer test procedures ☐ Imaging study
☐ Study of genetic markers ☐ Research interview ☐ Telemedical study
☐ Blood sampling for medical research purposes ☐ Other one

5.10
[ ]

5.11 Did you give permission to participate in a research project for your supervised person?
☐ Yes ☐ No

5.12 Who made the decision
☐ Exclusively you as the guardian ☐ The supervised person alone ☐ Together with the supervised person
☐ You after consultation with family members of the supervised person

5.13 Was the supervised person satisfied with your decision?
☐ Yes ☐ No ☐ No accurate assessment

5. Specific questions about research I [Fortsetzung]

5.14 What you be willing to give your permission for participation in resaerch projects for your supervised person in principle?

Taking into account the reason for care and the severity of the manifestation of any disease present, if this constitutes the reason for care

- ☐ Yes
- ☐ No

5.15 Who would likely make the decision?

- ☐ Exclusively you as the guardian
- ☐ The supervised person alone
- ☐ Together with the supervised person
- ☐ You after consultation with family members of the supervised person

5.16 For what kind of study could you imagine giving consent for your supervised person in general?

Taking into account the reason for care and the severity of the manifestation of any disease present, if it is the reason for care.

- ☐ Computer test procedures
- ☐ Imaging study
- ☐ Study for genetic markers
- ☐ Research interview
- ☐ Telemedical study
- ☐ Blood sampling for research purposes
- ☐ Others
- ☐ No participation

5.17

____

5.18 What are your motives agreeing to participate in medical research projects for your supervised person?
Multiple choice possible

- ☐ Hope for personal benefit
- ☐ Last hope/desperation
- ☐ Gain new knowledge to help further generations
- ☐ Gain new knowledge to help other patients
- ☐ Trust in doctors/researchers/ scientists
- ☐ Others

5.19

____

5.20 What are your motives <u>not</u> agreeing to participate in medical research projects for your supervised person?
Multiple choice possible

- ☐ Incalculable risk
- ☐ Too much burden on supervised person
- ☐ Too much time required
- ☐ No direct benefit for the supervised person
- ☐ Own illness to advanced
- ☐ No sense recognized in the study
- ☐ Incomprehensible methods
- ☐ Others

5.21

____

5.22 Would you override the supposed wishes of the supervised person if you think that participation in the relevant study would benefit this person?

- ☐ Yes
- ☐ No

6. Note

6.1 All the following questions please refer to the 2nd person you have chosen.
- ☐ OK

7. Specific questions about research II

7.1 What is the reason for the supervision?
- ☐ Intellectual disability
- ☐ Dementia
- ☐ Schizophrenia
- ☐ Mental illness except schizophrenia
- ☐ Neurological deficits
- ☐ Neglect
- ☐ Low intelligence
- ☐ Others

7.2

7.3 What domains are under supervision?
Multiple choice possible
- ☐ Financial management
- ☐ Healtcare matters
- ☐ Residential matters
- ☐ Housing matters
- ☐ Administrative matters
- ☐ Others

7.4

7.5 What is the age of the person you are supervising?

(In case of a request for study participation for this person, please indicate the age at the time of the request).
- ☐ under 30
- ☐ 30-40
- ☐ 31-40
- ☐ 41-50
- ☐ 51-60
- ☐ 61-70
- ☐ 71-80
- ☐ 81-90
- ☐ over 90

7.6 Since when you supervise this person? (in years)
- ☐ under 1
- ☐ 1-3
- ☐ 4-6
- ☐ 7-9
- ☐ 10 or more

7.7 How close is the relationship with this person?
- ☐ emotional
- ☐ Friendly
- ☐ Factual
- ☐ No accurate assessment

7.8 Should you made a decision in the past for this person to participate or not in research project?
- ☐ Yes
- ☐ No

7.9 What kind of study was it?
- ☐ Pharmaceutical study
- ☐ Computer test procedures
- ☐ Imaging study
- ☐ Study for genetic markers
- ☐ Research interview
- ☐ Telemedical study
- ☐ Blood sampling for research purposes
- ☐ Others

7.10

7.11 Did you give permission to participate in a research project for your supervised person?
- ☐ Yes
- ☐ No

7.12 Who made the decision?
- ☐ Exclusively you as the guardian
- ☐ The supervised person alone
- ☐ Together with the supervised person
- ☐ You after consultation with family members of the supervised person

7.13 Was the supervised person satisfied with your decision?
- ☐ Yes
- ☐ No
- ☐ No accurate assessment

7.14 What you be willing to give your permission for participation in resaerch projects for your supervised person in principle?

Taking into account the reason for care and the severity of the manifestation of any disease present, if this constitutes the reason for care
- ☐ Yes
- ☐ No

7.15 Who would likely make the decision?
- ☐ Exclusively you as the guardian
- ☐ The supervised person alone
- ☐ Together with the supervised person
- ☐ You after consultation with family members of the supervised person

7. Specific questions about research II [Fortsetzung]

7.16 For what kind of study could you imagine giving consent for your supervised person in general?

Taking into account the reason for care and the severity of the manifestation of any disease present, if it is the reason for care.

- ☐ Computer test procedures
- ☐ Imaging study
- ☐ Study for genetic markers
- ☐ Research interview
- ☐ Telemedical study
- ☐ Blood sampling for research purposes
- ☐ Others
- ☐ No participation

7.17

[ ]

7.18 What are your motives agreeing to participate in medical research projects for your supervised person?
Multiple choice possible

- ☐ Hope for personal benefit
- ☐ Last hope/desperation
- ☐ Gain new knowledge to help further generations
- ☐ Gain new knowledge to help other patients
- ☐ Trust in doctors/researchers/ scientists
- ☐ Others

7.19

[ ]

7.20 What are your motives not agreeing to participate in medical research projects for your supervised person?
Multiple choice possible

- ☐ Incalculable risk
- ☐ Too much burden on the supervised person
- ☐ Too much time required
- ☐ No direct benefit for the supervised person
- ☐ Illness to advanced
- ☐ No sense recognized in the study
- ☐ Incomprehensible methods
- ☐ Others

7.21

[ ]

7.22 Would you override the supposed wishes of the supervised person if you think that participation in the relevant study would benefit this person?

- ☐ Yes
- ☐ No

8. Note

8.1 All the following questions please refer to the 3rd person you have chosen.
- ☐ OK

9. Specific questions about research III

9.1 What is the reason for the supervision?
- ☐ Intellectual disability
- ☐ Dementia
- ☐ Schizophrenia
- ☐ Mental illness except schizophrenia
- ☐ Neurological deficits
- ☐ Neglect
- ☐ Low intelligence
- ☐ Others

9.2

9.3 What domains are under supervision?
Multiple choice possible
- ☐ Financial management
- ☐ Healthcare matters
- ☐ Residational matters
- ☐ Housing matters
- ☐ Administrative matters
- ☐ Others

9.4

9.5 What is the age of the person you are supervising?

(In case of a request for study participation for this person, please indicate the age at the time of the request).
- ☐ under 30
- ☐ 30-40
- ☐ 31-40
- ☐ 41-50
- ☐ 51-60
- ☐ 61-70
- ☐ 71-80
- ☐ 81-90
- ☐ over 90

9.6 Since when you supervise this person? (in years)
- ☐ under 1
- ☐ 1-3
- ☐ 4-6
- ☐ 7-9
- ☐ 10 or more

9.7 How close is the relationship with this person?
- ☐ Emotional
- ☐ Friendly
- ☐ Factual
- ☐ No accurat assessment

9.8 Should you made a decision in the past for this person to participate or not in research project?
- ☐ Yes
- ☐ No

9.9 What kind of study was it?
- ☐ Pharmaceutical study
- ☐ Computer test procedure
- ☐ Imaging study
- ☐ Study of genetic markers
- ☐ Research interview
- ☐ Telemedical study
- ☐ Blood sampling for research purposes
- ☐ Others

9.10

9.11 Did you give permission to participate in a research project for your supervised person?
- ☐ Yes
- ☐ No

9.12 Who made the decision?
- ☐ Exclusively you as the guardian
- ☐ The supervised person alone
- ☐ Together with the supervised person
- ☐ You after consultation with family members of the supervised person

9.13 Was the supervised person satisfied with your decision?
- ☐ Yes
- ☐ No
- ☐ No accurat assessment

9.14 What you be willing to give your permission for participation in resaerch projects for your supervised person in principle?

Taking into account the reason for care and the severity of the manifestation of any disease present, if this constitutes the reason for care.
- ☐ Yes
- ☐ No

9.15 Who would likely make the decision?
- ☐ Exclusively you as the guardian
- ☐ The supervised person alone
- ☐ Together with the supervised person
- ☐ You after consultation with family members of the supervised person

MUSTER

9. Specific questions about research III [Fortsetzung]

9.16 For what kind of study could you imagine giving consent for your supervised person in general?

Taking into account the reason for care and the severity of the manifestation of any disease present, if it is the reason for care.

- ☐ Computert test procedures
- ☐ Imaging study
- ☐ Study of genetic markers
- ☐ Research interview
- ☐ Telemedical study
- ☐ Blood sampling for research purposes
- ☐ Others
- ☐ No participation

9.17

[ ]

9.18 What are your motives agreeing to participate in medical research projects for your supervised person?
Multiple choice possible

- ☐ Hope of personal benefit
- ☐ last hope/desperation
- ☐ Gain new knowledge to help further generations
- ☐ Gain new knowledge to help other patients
- ☐ Trust in doctors/researchers/scientists
- ☐ Others

9.19

[ ]

9.20 What are your motives <u>not</u> agreeing to participate in medical research projects for your supervised person?
Multiple choice possible

- ☐ Incalculable risk
- ☐ Too much burden on the supervised person
- ☐ Too much time required
- ☐ No direct benefit
- ☐ Illness to advanced
- ☐ No sense recognized in the study
- ☐ Incomprehensible methods
- ☐ Others

9.21

[ ]

9.22 Would you override the supposed wishes of the supervised person if you think that participation in the relevant study would benefit this person?

- ☐ Yes
- ☐ No

MUSTER
