# Supplementary material for: Attitudes of legal guardians and legally supervised persons with and without previous research experience towards participation in research projects: A quantitative cross-sectional study
Source: PLoS One. 2021 Sep 15;16(9):e0256689. doi: 10.1371/journal.pone.0256689 (PMC8443074; doi:10.1371/journal.pone.0256689)
Supplement: S2 File — (PDF) [file pone.0256689.s002.pdf]

MUSTER

Universitätsmedizin Greifswald
Institut für Community Medicine, Abteilung Betreuerstudie 2019

Universitätsmedizin GREIFSWALD

Bitte so markieren: ☐ ☒ ☐ ☐ ☐ Bitte verwenden Sie einen Kugelschreiber oder nicht zu starken Filzstift. Dieser Fragebogen wird maschinell erfasst.

Korrektur: ☐ ■ ☐ ☒ ☐ Bitte beachten Sie im Interesse einer optimalen Datenerfassung die links gegebenen Hinweise beim Ausfüllen.

1. Aufklärung

Sehr geehrte Betreuerin, sehr geehrter Betreuer,

vielen Dank, dass Sie sich die Zeit nehmen den nachfolgenden Fragebogen zu beantworten.

Mit Ihrer Hilfe möchte wir, Wissenschaftler der Universitätsmedizin Greifswald, untersuchen, welche Gründe aus Ihrer Sicht für bzw. gegen eine Teilnahme an wissenschaftlichen Studien im Bereich Gesundheit und Versorgung sprechen.

In Zukunft wird es immer wichtiger, dass auch Menschen mit einer gesetzlichen Betreuung an Forschungsprojekten teilnehmen können. Das ist wichtig, weil auch Menschen mit einer Betreuung an Fortschritten in der medizinischen Versorgung teilhaben können müssen. In diesem Fragebogen werden Ihnen zunächst einige Fragen zu Ihrer Betreuungssituation gestellt. Danach möchten wir etwas über Ihre Gründe zur Zustimmung oder Ablehnung einer Teilnahme an wissenschaftlichen Projekten erfahren.

Es gibt bei der Beantwortung der Fragen kein „richtig“ oder „falsch“, nur Ihre persönliche Meinung zählt. Die Beantwortung der nachfolgenden Fragen wird etwa 10 Minuten in Anspruch nehmen.

Ihre Daten werden von uns streng vertraulich behandelt. Alle Daten unterliegen dem Datenschutz und werden ausschließlich anonymisiert verarbeitet und nicht an Dritte weitergegeben. Ihre Daten dienen ausschließlich dem wissenschaftlichen Zweck dieser Befragung und werden anschließend nicht weiterverwendet. Die Teilnahme an der Befragung ist freiwillig.

Grundsätzlich sind Entscheidungen des Betreuers so zu treffen, dass dem Wohle der betreuten Person entsprochen und diese nicht bevormundet wird. Wichtige Angelegenheiten sind in Absprache mit der betreuten Person zu treffen, sofern dadurch kein Nachteil für die betreute Person entsteht. (§1901 Abs. 3 BGB)

Sollten Sie Fragen zu dieser Befragung haben, kontaktieren Sie uns gerne per E-Mail (betreuerstudie@uni-greifswald.de).

1.1

☐ Ich habe den obigen Text zur Kenntnis genommen und bin mit der Teilnahme an der Studie einverstanden.

2. Soziodemographische Fragen

2.1 Bitte geben Sie Ihr Alter an (in Jahren)

☐ unter 30 ☐ 30-40 ☐ 41-50
☐ 51-60 ☐ über 60

2.2 Bitte geben Sie Ihr Geschlecht an

☐ männlich ☐ weiblich ☐ divers

2.3 Wie ist Ihr Familienstand?

☐ ledig ☐ verheiratet ☐ dauernd getrennt lebend
☐ geschieden ☐ verwitwet

2.4 Was ist Ihr höchster Bildungsabschluss

☐ kein Schulabschluss ☐ Berufsreife ☐ mittlere Reife
☐ (Fach-)Hochschulreife ☐ Hochschulabschluss ☐ Promotion
☐ Habilitation

2.5 Was sind Sie von Beruf

[ ]

2.6 Welche Betreuungsform führen Sie aus?

☐ ehrenamtlich ☐ beruflich

MUSTER

2. Soziodemographische Fragen [Fortsetzung]

2.7 Wie viele Personen betreuen Sie aktuell?

- ☐ 1
- ☐ 2
- ☐ 3
- ☐ 4-6
- ☐ 7-9
- ☐ 10-12
- ☐ 13-15
- ☐ 16-18
- ☐ 19-21
- ☐ mehr als 21

2.8 Wie stehen Sie der medizinischen Forschung gegenüber?

- ☐ offene Haltung
- ☐ neutrale Haltung
- ☐ geschlossene Haltung
- ☐ noch keine Gedanken darüber gemacht

2.9 Bei welcher Art von Studie könnten Sie sich vorstellen teilzunehmen?
Mehrfachnennung möglich

- ☐ Computertestverfahren (bspw. Test zur Feststellung des IQ oder Reaktionsvermögens)
- ☐ Bildgebende Studien (bspw. MRT-Untersuchung zur Darstellung veränderter Hirnstrukturen bei Demenzpatienten)
- ☐ Untersuchung von genetischen Markern (wichtig bei Krankheiten mit erblicher Komponente und zur Erkennung verschiedener Erkrankungen)
- ☐ Interview
- ☐ Telemedizinische Studie (bspw. Führung von Arzt-Patienten-Gesprächen via Video-Telefonat oder Kontaktaufnahme via SMS)
- ☐ Blutentnahme zu Zwecken der medizinischen Forschung
- ☐ Sonstige
- ☐ keine Teilnahme

2.10

[ ]

3. Anmerkung

3.1 Da Sie mehr als eine Person betreuen wählen Sie bitte 2 bzw. 3 von Ihren betreuten Personen aus. Für diese Personen beantworten Sie die folgenden Fragen bitte nacheinander. Durch entsprechende Hinweise werden Sie darauf aufmerksam gemacht, ab welchem Zeitpunkt die Fragen für die 2. bzw. 3. Person zu beantworten sind.

Wenn Sie in der Vergangenheit schon einmal zu einer Teilnahme an einem wissenschaftlichen Forschungsprojekt für eine Ihrer betreuten Personen aufgefordert wurden, so beantworten Sie bitte die Fragen bezogen auf diese Person.

Falls in der Vergangenheit keine Aufforderung zur Teilnahme vorlag wählen Sie bitte 2 bzw. 3 der von Ihnen betreuten Personen mit möglichst unterschiedlichen Betreuungsgründen aus.

☐ OK

4. Hinweis I

4.1 Alle nachfolgenden Fragen bitte bezogen auf die 1. von Ihnen auserwählte Person beziehen.

☐ OK

5. Spezifische Fragen zur Forschung I

5.1 Aus welchem Grund besteht das Betreuungsverhältnis?

☐ Geistige Behinderung ☐ Demenz ☐ Schizophrenie
☐ Andere psychische Erkrankung ☐ Neurologische Erkrankung ☐ Verwahrlosung
☐ Geringe Intelligenz ☐ Sonstige

5.2

____

5.3 Für welchen Bereich besteht die Betreuung?
Mehrfachnennung möglich

☐ Vermögensregelung ☐ Gesundheitsfürsorge ☐ Heimangelegenheiten
☐ Wohnungsangelegenheiten ☐ Behördenangelegenheiten ☐ Sonstige

5.4

____

5.5 Wie alt ist diese von Ihnen betreute Person?

(Im Falle einer Anfrage zur Studienteilnahme für diese Person bitte das Alter zu dem Zeitpunkt der Anfrage angeben)

☐ unter 30 ☐ 30-40 ☐ 31-40
☐ 41-50 ☐ 51-60 ☐ 61-70
☐ 71-80 ☐ 81-90 ☐ über 90

5.6 Seit wann besteht das Betreuungsverhältnis für diese von Ihnen betreute Person? (in Jahren)

☐ unter 1 ☐ 1-3 ☐ 4-6
☐ 7-9 ☐ 10 oder länger

5.7 Wie häufig haben Sie Kontakt zu dieser von Ihnen betreuten Person? (pro Woche)

☐ weniger als 1-mal ☐ 1- bis 2-mal ☐ 3- bis 4-mal
☐ 5- bis 6-mal ☐ 7-mal

5.8 Wie haben Sie am häufigsten Kontakt zu dieser von Ihnen betreuten Person?

☐ via Telefon ☐ via SMS/ WhatsApp ☐ via E-Mail
☐ persönliche Besuche ☐ Sonstige

5.9 Wie würden Sie das Verhältnis zu der von Ihnen betreuten Person beschreiben?

☐ emotional ☐ freundschaftlich ☐ sachlich
☐ weiß nicht

5.10 Wurden Sie in der Vergangenheit aufgefordert für diese von Ihnen betreute Person zu entscheiden, ob diese Person an einer wissenschaftlichen Studie teilnehmen soll(te)?

☐ ja ☐ nein

5. Spezifische Fragen zur Forschung I [Fortsetzung]

5.11 Um welche Art von Studie handelte es sich?

- ☐ Arzneimittelstudie
- ☐ Computertestverfahren (bspw. Test zur Feststellung des IQ oder Reaktionsvermögens)
- ☐ Bildgebende Studie (bspw. MRT-Untersuchung zur Darstellung veränderter Hirnstrukturen bei Demenzpatienten)
- ☐ Untersuchung genetischer Marker (wichtig bei Krankheiten mit erblicher Komponente und zur Erkennung verschiedener Krankheiten)
- ☐ Interview
- ☐ Telemedizinische Studie (bspw. Führung von Arzt-Patienten-Gesprächen via Video-Telefonat oder Kontaktaufnahme via SMS)
- ☐ Blutentnahme zu Zwecken medizinischer Forschung
- ☐ Sonstige

5.12

[ ]

5.13 Haben Sie der Teilnahme für die von Ihnen betreuten Person zugestimmt?

- ☐ ja
- ☐ nein

5.14 Wer hat die Entscheidung getroffen?

- ☐ Ausschließlich Sie als Betreuer
- ☐ Der Betreute allein
- ☐ Gemeinsam
- ☐ Sie nach Rücksprache mit Familienangehörigen der von Ihnen betreuten Person

5.15 War der Betreute mit Ihrer Entscheidung einverstanden?

- ☐ ja
- ☐ nein
- ☐ weiß nicht

5.16 Wären Sie grundsätzlich bereit Ihre Zustimmung zur Teilnahme an einer wissenschaftlichen Studie für diese von Ihnen betreute Person zu erteilen?

Unter Berücksichtigung des Betreuungsgrundes und des Schweregrades der Ausprägung einer eventuell vorliegenden Krankheit, wenn diese den Betreuungsgrund darstellt

- ☐ ja
- ☐ nein

5.17 Wer würde die Entscheidung voraussichtlich treffen?

- ☐ Ausschließlich Sie als Betreuer
- ☐ Der Betreute allein
- ☐ Gemeinsam
- ☐ Sie nach Rücksprache mit Familienangehörigen der von Ihnen betreuten Person

5.18 Für welche Art von Studie könnten Sie sich grundsätzlich vorstellen die Einwilligung zur Teilnahme für diese von Ihnen betreute Person zu geben?
Mehrfachnennung möglich

Unter Berücksichtigung des Betreuungsgrundes und des Schweregrades der Ausprägung einer eventuell vorliegenden Krankheit, wenn diese den Betreuungsgrund darstellt.

- ☐ Computerttestverfahren (bspw. Test zur Feststellung des IQ oder Reaktionsvermögens)
- ☐ Bildgebende Studie (bspw. MRT-Untersuchung zur Darstellung veränderter Hirnstrukturen bei Demenzpatienten)
- ☐ Untersuchung genetischer Marker (wichtig bei Krankheiten mit erblicher Komponente und zur Erkennung verschiedener Erkrankungen)
- ☐ Interview
- ☐ Telemedizinische Studie (bspw. Führung von Arzt-Patienten-Gesprächen via Video-Telefonat oder Kontaktaufnahme via SMS)
- ☐ Blutentnahme zu Zwecken der medizinischen Forschung
- ☐ Sonstige
- ☐ keine Teilnahme

5.19

[ ]

5. Spezifische Fragen zur Forschung I [Fortsetzung]

5.20 Was war der Grund bzw. was würden Sie als Grund für Ihre Zustimmung zur Teilnahme an einer wissenschaftliche Studie sehen?
Mehrfachnennung möglich

- ☐ Hoffnung auf persönlichen Nutzen
- ☐ Letzte Hoffnung/ Verzweiflung
- ☐ Gewinn neuer Erkenntnisse um Nachkommen in Zukunft helfen zu können
- ☐ Gewinn neuer Erkenntnisse um anderen Leidenden helfen zu können
- ☐ Vertrauen in Forscher/ Wissenschaftler/ Ärzte
- ☐ Sonstige

5.21

[ ]

5.22 Was war der Grund bzw. was würden Sie als Grund für Ihre Ablehnung zur Teilnahme an einer wissenschaftlichen Studie sehen?
Mehrfachnennung möglich

- ☐ Risiko nicht abschätzbar
- ☐ Zu große Belastung für den Patienten
- ☐ Zu großer zeitlicher Aufwand
- ☐ Kein direkter Nutzen für den Betreuten
- ☐ Krankheit zu weit fortgeschritten
- ☐ Keine Sinnhaftigkeit in der Studie erkannt
- ☐ Methodik unverständlich
- ☐ Sonstige

5.23

[ ]

5.24 Würden Sie sich über den vermeintlichen Wunsch dieser von Ihnen betreuten Person hinwegsetzen, wenn Sie der Meinung sind dass die Teilnahme an der entsprechenden Studie einen Vorteil für diese betreute Person bringen würde?

- ☐ ja
- ☐ nein

MUSTER

6. Hinweis II

6.1 Alle nachfolgenden Fragen bitte bezogen auf die 2. von Ihnen auserwählte Person beziehen.
- ☐ OK

7. Spezifische Fragen zur Forschung II

7.1 Aus welchem Grund besteht das Betreuungsverhältnis?
- ☐ Geistige Behinderung
- ☐ Demenz
- ☐ Schizophrenie
- ☐ Andere psychische Erkrankung
- ☐ Neurologische Erkrankung
- ☐ Verwahrlosung
- ☐ Geringe Intelligenz
- ☐ Sonstige

7.2

[ ]

7.3 Für welchen Bereich besteht die Betreuung?
Mehrfachnennung möglich
- ☐ Vermögensregelung
- ☐ Gesundheitsfürsorge
- ☐ Heimangelegenheiten
- ☐ Wohnungsangelegenheiten
- ☐ Behördenangelegenheiten
- ☐ Sonstige

7.4

[ ]

7.5 Wie alt ist diese von Ihnen betreute Person?

(Im Falle einer Anfrage zur Studienteilnahme für diese Person bitte das Alter zu dem Zeitpunkt der Anfrage angeben)
- ☐ unter 30
- ☐ 30-40
- ☐ 31-40
- ☐ 41-50
- ☐ 51-60
- ☐ 61-70
- ☐ 71-80
- ☐ 81-90
- ☐ über 90

7.6 Seit wann besteht das Betreuungsverhältnis für diese von Ihnen betreute Person? (in Jahren)
- ☐ unter 1
- ☐ 1-3
- ☐ 4-6
- ☐ 7-9
- ☐ 10 oder länger

7.7 Wie häufig haben Sie Kontakt zu dieser von Ihnen betreuten Person? (pro Woche)
- ☐ weniger als 1-mal
- ☐ 1- bis 2-mal
- ☐ 3- bis 4-mal
- ☐ 5- bis 6-mal
- ☐ 7-mal

7.8 Wie haben Sie am häufigsten Kontakt zu dieser von Ihnen betreuten Person?
- ☐ via Telefon
- ☐ via SMS/ WhatsApp
- ☐ via E-Mail
- ☐ persönliche Besuche
- ☐ Sonstige

7.9 Wie würden Sie das Verhältnis zu der von Ihnen betreuten Person beschreiben?
- ☐ emotional
- ☐ freundschaftlich
- ☐ sachlich
- ☐ weiß nicht

7.10 Wurden Sie in der Vergangenheit aufgefordert für diese von Ihnen betreute Person zu entscheiden, ob diese Person an einer wissenschaftlichen Studie teilnehmen soll(te)?
- ☐ ja
- ☐ nein

7.11 Um welche Art von Studie handelte es sich?
- ☐ Arzneimittelstudie
- ☐ Computertestverfahren (bspw. Test zur Feststellung des IQ oder Reaktionsvermögens)
- ☐ Bildgebende Studie (bspw. MRT-Untersuchung zur Darstellung veränderter Hirnstrukturen bei Demenzpatienten)
- ☐ Untersuchung genetischer Marker (wichtig bei Krankheiten mit erblicher Komponente und zur Erkennung verschiedener Erkrankungen)
- ☐ Interview
- ☐ Telemedizinische Studie (bspw. Führung von Arzt-Patienten-Gesprächen via Video-Telefonat oder Kontaktaufnahme via SMS)
- ☐ Blutentnahme zu Zwecken medizinischer Forschung
- ☐ Sonstige

7.12

[ ]

7.13 Haben Sie der Teilnahme für die von Ihnen betreuten Person zugestimmt?
- ☐ ja
- ☐ nein

MUSTER

7. Spezifische Fragen zur Forschung II [Fortsetzung]

7.14 Wer hat die Entscheidung getroffen?
- ☐ Ausschließlich Sie als Betreuer
- ☐ Der Betreute allein
- ☐ Gemeinsam
- ☐ Sie nach Rücksprache mit Familienangehörigen der von Ihnen betreuten Person

7.15 War der Betreute mit Ihrer Entscheidung einverstanden?
- ☐ ja
- ☐ nein
- ☐ weiß nicht

7.16 Wären Sie grundsätzlich bereit Ihre Zustimmung zur Teilnahme an einer wissenschaftlichen Studie für diese von Ihnen betreute Person zu erteilen?

Unter Berücksichtigung des Betreuungsgrundes und des Schweregrades der Ausprägung einer eventuell vorliegenden Krankheit, wenn diese den Betreuungsgrund darstellt

- ☐ ja
- ☐ nein

7.17 Wer würde die Entscheidung voraussichtlich treffen?
- ☐ Ausschließlich Sie als Betreuer
- ☐ Der Betreute allein
- ☐ Gemeinsam
- ☐ Sie nach Rücksprache mit Familienangehörigen der von Ihnen betreuten Person

7.18 Für welche Art von Studie könnten Sie sich grundsätzlich vorstellen die Einwilligung zur Teilnahme für diese von Ihnen betreute Person zu geben?
Mehrfachnennung möglich

Unter Berücksichtigung des Betreuungsgrundes und des Schweregrades der Ausprägung einer eventuell vorliegenden Krankheit, wenn diese den Betreuungsgrund darstellt.

- ☐ Computerttestverfahren (bspw. Test zur Feststellung des IQ oder Reaktionsvermögens)
- ☐ Bildgebende Studie (bspw. MRT-Untersuchung zur Darstellung veränderter Hirnstrukturen bei Demenzpatienten)
- ☐ Untersuchung genetischer Marker (wichtig bei Krankheiten mit erblicher Komponente und zur Erkennung verschiedener Erkrankungen)
- ☐ Interview
- ☐ Telemedizinische Studie (bspw. Führung von Arzt-Patienten-Gesprächen via Video-Telefonat oder Kontaktaufnahme via SMS)
- ☐ Blutentnahme zu Zwecken der medizinischen Forschung
- ☐ Sonstige
- ☐ keine Teilnahme

7.19

[ ]

7.20 Was war der Grund bzw. was würden Sie als Grund für Ihre Zustimmung zur Teilnahme an einer wissenschaftliche Studie sehen?
Mehrfachnennung möglich
- ☐ Hoffnung auf persönlichen Nutzen
- ☐ Letzte Hoffnung/ Verzweiflung
- ☐ Gewinn neuer Erkenntnisse um Nachkommen in Zukunft helfen zu können
- ☐ Gewinn neuer Erkenntnisse um anderen Leidenden helfen zu können
- ☐ Vertrauen in Forscher/ Wissenschaftler/ Ärzte
- ☐ Sonstige

7.21

[ ]

7.22 Was war der Grund bzw. was würden Sie als Grund für Ihre Ablehnung zur Teilnahme an einer wissenschaftlichen Studie sehen?
Mehrfachnennung möglich
- ☐ Risiko nicht abschätzbar
- ☐ Zu große Belastung für den Patienten
- ☐ Zu großer zeitlicher Aufwand
- ☐ Kein direkter Nutzen für den Betreuten
- ☐ Krankheit zu weit fortgeschritten
- ☐ Keine Sinnhaftigkeit in der Studie erkannt
- ☐ Methodik unverständlich
- ☐ Sonstige

7.23

[ ]

MUSTER

7. Spezifische Fragen zur Forschung II [Fortsetzung]

7.24 Würden Sie sich über den vermeintlichen Wunsch dieser von Ihnen betreuten Person hinwegsetzen, wenn Sie der Meinung sind dass die Teilnahme an der entsprechenden Studie einen Vorteil für diese betreute Person bringen würde?

☐ ja ☐ nein

MUSTER

MUSTER

8. Hinweis III

8.1 Alle nachfolgenden Fragen bitte bezogen auf die 3. von Ihnen auserwählte Person beziehen.
- ☐ OK

9. Spezifische Fragen zur Forschung III

9.1 Aus welchem Grund besteht das Betreuungsverhältnis?
- ☐ Geistige Behinderung
- ☐ Demenz
- ☐ Schizophrenie
- ☐ Andere psychische Erkrankung
- ☐ Neurologische Erkrankung
- ☐ Verwahrlosung
- ☐ Geringe Intelligenz
- ☐ Sonstige

9.2

9.3 Für welchen Bereich besteht die Betreuung?
Mehrfachnennung möglich
- ☐ Vermögensregelung
- ☐ Gesundheitsfürsorge
- ☐ Heimangelegenheiten
- ☐ Wohnungsangelegenheiten
- ☐ Behördenangelegenheiten
- ☐ Sonstige

9.4

9.5 Wie alt ist diese von Ihnen betreute Person?

(Im Falle einer Anfrage zur Studienteilnahme für diese Person bitte das Alter zu dem Zeitpunkt der Anfrage angeben)
- ☐ unter 30
- ☐ 30-40
- ☐ 31-40
- ☐ 41-50
- ☐ 51-60
- ☐ 61-70
- ☐ 71-80
- ☐ 81-90
- ☐ über 90

9.6 Seit wann besteht das Betreuungsverhältnis für diese von Ihnen betreute Person? (in Jahren)
- ☐ unter 1
- ☐ 1-3
- ☐ 4-6
- ☐ 7-9
- ☐ 10 oder länger

9.7 Wie häufig haben Sie Kontakt zu dieser von Ihnen betreuten Person? (pro Woche)
- ☐ weniger als 1-mal
- ☐ 1- bis 2-mal
- ☐ 3- bis 4-mal
- ☐ 5- bis 6-mal
- ☐ 7-mal

9.8 Wie haben Sie am häufigsten Kontakt zu dieser von Ihnen betreuten Person?
- ☐ via Telefon
- ☐ via SMS/ WhatsApp
- ☐ via E-Mail
- ☐ persönliche Besuche
- ☐ Sonstige

9.9 Wie würden Sie das Verhältnis zu der von Ihnen betreuten Person beschreiben?
- ☐ emotional
- ☐ freundschaftlich
- ☐ sachlich
- ☐ weiß nicht

9.10 Wurden Sie in der Vergangenheit aufgefordert für diese von Ihnen betreute Person zu entscheiden, ob diese Person an einer wissenschaftlichen Studie teilnehmen soll(te)?
- ☐ ja
- ☐ nein

9.11 Um welche Art von Studie handelte es sich?
- ☐ Arzneimittelstudie
- ☐ Computertestverfahren (bspw. Test zur Feststellung des IQ oder Reaktionsvermögens)
- ☐ Bildgebende Studie (bspw. MRT-Untersuchung zur Darstellung veränderter Hirnstrukturen bei Demenzpatienten)
- ☐ Untersuchung genetischer Marker (wichtig bei Krankheiten mit erblicher Komponente und zur Erkennung verschiedener Erkrankungen)
- ☐ Interview
- ☐ Telemedizinische Studie (bspw. Führung von Arzt-Patienten-Gesprächen via Video-Telefonat oder Kontaktaufnahme via SMS)
- ☐ Blutentnahme zu Zwecken medizinischer Forschung
- ☐ Sonstige

9.12

9.13 Haben Sie der Teilnahme für die von Ihnen betreuten Person zugestimmt?
- ☐ ja
- ☐ nein

MUSTER

9. Spezifische Fragen zur Forschung III [Fortsetzung]

9.14 Wer hat die Entscheidung getroffen?
- ☐ Ausschließlich Sie als Betreuer
- ☐ Der Betreute allein
- ☐ Gemeinsam
- ☐ Sie nach Rücksprache mit Familienangehörigen der von Ihnen betreuten Person

9.15 War der Betreute mit Ihrer Entscheidung einverstanden?
- ☐ ja
- ☐ nein
- ☐ weiß nicht

9.16 Wären Sie grundsätzlich bereit Ihre Zustimmung zur Teilnahme an einer wissenschaftlichen Studie für diese von Ihnen betreute Person zu erteilen?

Unter Berücksichtigung des Betreuungsgrundes und des Schweregrades der Ausprägung einer eventuell vorliegenden Krankheit, wenn diese den Betreuungsgrund darstellt

- ☐ ja
- ☐ nein

9.17 Wer würde die Entscheidung voraussichtlich treffen?
- ☐ Ausschließlich Sie als Betreuer
- ☐ Der Betreute allein
- ☐ Gemeinsam
- ☐ Sie nach Rücksprache mit Familienangehörigen der von Ihnen betreuten Person

9.18 Für welche Art von Studie könnten Sie sich grundsätzlich vorstellen die Einwilligung zur Teilnahme für diese von Ihnen betreute Person zu geben?
Mehrfachnennung möglich

Unter Berücksichtigung des Betreuungsgrundes und des Schweregrades der Ausprägung einer eventuell vorliegenden Krankheit, wenn diese den Betreuungsgrund darstellt.

- ☐ Computerttestverfahren (bspw. Test zur Feststellung des IQ oder Reaktionsvermögens)
- ☐ Bildgebende Studie (bspw. MRT-Untersuchung zur Darstellung veränderter Hirnstrukturen bei Demenzpatienten)
- ☐ Untersuchung genetischer Marker (wichtig bei Krankheiten mit erblicher Komponente und zur Erkennung verschiedener Erkrankungen)
- ☐ Interview
- ☐ Telemedizinische Studie (bspw. Führung von Arzt-Patienten-Gesprächen via Video-Telefonat oder Kontaktaufnahme via SMS)
- ☐ Blutentnahme zu Zwecken der medizinischen Forschung
- ☐ Sonstige
- ☐ keine Teilnahme

9.19

[ ]

9.20 Was war der Grund bzw. was würden Sie als Grund für Ihre Zustimmung zur Teilnahme an einer wissenschaftliche Studie sehen?
Mehrfachnennung möglich
- ☐ Hoffnung auf persönlichen Nutzen
- ☐ Letzte Hoffnung/ Verzweiflung
- ☐ Gewinn neuer Erkenntnisse um Nachkommen in Zukunft helfen zu können
- ☐ Gewinn neuer Erkenntnisse um anderen Leidenden helfen zu können
- ☐ Vertrauen in Forscher/ Wissenschaftler/ Ärzte
- ☐ Sonstige

9.21

[ ]

9.22 Was war der Grund bzw. was würden Sie als Grund für Ihre Ablehnung zur Teilnahme an einer wissenschaftlichen Studie sehen?
Mehrfachnennung möglich
- ☐ Risiko nicht abschätzbar
- ☐ Zu große Belastung für den Patienten
- ☐ Zu großer zeitlicher Aufwand
- ☐ Kein direkter Nutzen für den Betreuten
- ☐ Krankheit zu weit fortgeschritten
- ☐ Keine Sinnhaftigkeit in der Studie erkannt
- ☐ Methodik unverständlich
- ☐ Sonstige

9.23

[ ]

MUSTER

9. Spezifische Fragen zur Forschung III [Fortsetzung]

9.24 Würden Sie sich über den vermeintlichen Wunsch dieser von Ihnen betreuten Person hinwegsetzen, wenn Sie der Meinung sind dass die Teilnahme an der entsprechenden Studie einen Vorteil für diese betreute Person bringen würde?

☐ ja ☐ nein

MUSTER
