# Supplementary material for: Attitudes of legal guardians and legally supervised persons with and without previous research experience towards participation in research projects: A quantitative cross-sectional study
Source: PLoS One. 2021 Sep 15;16(9):e0256689. doi: 10.1371/journal.pone.0256689 (PMC8443074; doi:10.1371/journal.pone.0256689)
Supplement: S3 File — (PDF) [file pone.0256689.s003.pdf]

EvaSys	Questionnaire legally supervised persons	Electric Paper

University Medicine Greifswald

Institute for Community Medicine, Department Guardianship study 2019

Bitte so markieren: ☐ ☒ ☐ ☐ ☐
Korrektur: ☐ ■ ☐ ☒ ☐

1. Declaration of consent

Dear Participant,

Thank you for taking the time to answer the following questionnaire.

With your help, we, scientists of the University Medicine Greifswald, would like to examine your motives for or against participation in medical research projects.

In the future, it will become increasingly important that people with legal care can also participate in medical research projects. This is important because people with care also need to be able to participate in advances in medical care. In this questionnaire, you will first be asked some questions about your guardianship. Then we would like to know more about your motives for or against participation in research projects.

There is no "right" or "wrong" in answering the questions; only your opinion matters. Answering the questionnaire will take about 8 minutes.

We will treat your data with strict confidentiality. All data are subject to data protection and will only be processed anonymously and will not be passed on to third parties. Your data will be used exclusively for the scientific purpose of this study and will not be used for any other purpose afterward. Participation in the survey is voluntary.

If you have any questions about this survey, please feel free to contact us via e-mail (betreuerstudie@uni-greifswald.de).

1.1

☐ I have taken note of the text above and agree to participate in this study.

2. Sociodemographic questions

2.1 Please state your age (in years)
- ☐ under 30
- ☐ 30-40
- ☐ 41-50
- ☐ 51-60
- ☐ 61-70
- ☐ 71-80
- ☐ 81-90
- ☐ over 90

2.2 Please state your gender
- ☐ Female
- ☐ Male
- ☐ Diverse

2.3 What is your family status?
- ☐ Unmarried
- ☐ Married
- ☐ Living permanently apart
- ☐ Divorced
- ☐ Widowed

2.4 What are you by profession?

[ ]

2.5 What is your current living situation?
- ☐ Alone
- ☐ With Others
- ☐ With a support of a nursing service
- ☐ In a nursing home
- ☐ Other

2.6

[ ]

3. Questions about your supervision

3.1 Since when you are supervised by a legal guardian? (in years)
- ☐ under 1
- ☐ 1-3
- ☐ 4-6
- ☐ 7-9
- ☐ 10 or more

3.2 Since when you are supervised by the current legal guardian? (in years)
- ☐ under 1
- ☐ 1-3
- ☐ 4-6
- ☐ 7-9
- ☐ 10 or more

3.3 What is the reason for the supervision?

[ ]

MUSTER

3. Questions about your supervision [Fortsetzung]

3.4 What domains are under supervision?
Multiple choice possible

- ☐ Financial management
- ☐ Healthcare matters
- ☐ Residential matters
- ☐ Housing matters
- ☐ Administrative matters
- ☐ Others
- ☐ No accurate assessment

3.5

3.6 How close is the relationship with your guardian?

- ☐ Emotional
- ☐ Friendly
- ☐ Factual
- ☐ Other

3.7

4. Questions about medical research projects

4.1 Have you ever been asked to participate in a medical research project in the past?

- ☐ Yes
- ☐ No

4.2 What kind of study was it?

- ☐ Pharmaceutical study
- ☐ Computer test procedures (e.g. test to determine IQ or reaction time)
- ☐ Imaging study (e.g. MRI examination to visualize altered brain structures in dementia patients)
- ☐ Study of genetic markers (important in the context with a hereditary component)
- ☐ Research interview
- ☐ Blood sampling for research purposes
- ☐ telemedical study (e.g. conducting doctor-patient conservations via video callm or contact via SMS)
- ☐ Others

4.3

4.4 Have you agreed to participate?

- ☐ Yes
- ☐ No

4.5 Who had made the decision?

- ☐ You on your own
- ☐ Your legal guardian
- ☐ You together with the legal guardian
- ☐ You together with family memebers
- ☐ Others

4.6

4.7 Would you be willing to participate in a medical research project in principle?

- ☐ Yes
- ☐ No

4.8 In which kind of study could you imagine participating?
Multiple choice possible

- ☐ Pharmaceutical study
- ☐ Computer test procedures (e.g. test to determine the IQ oder reaction time)
- ☐ Imaging study (e.g. MRI examination to visualize altered brain structures in dementia patients)
- ☐ Study of genetic markers (important in the context with a hereditary component)
- ☐ Research interview
- ☐ Blood sampling for medical research purposes
- ☐ Telemedical study (e.g. Führung von Arzt-Patienten-Gesprächen via Video-Telefonat oder Kontaktaufnahme via SMS)
- ☐ Others

4.9

4.10 Who would likely make the decision?

- ☐ You on your own
- ☐ Your legal guardian alone
- ☐ You together with your legal guardian
- ☐ You after consultation with your family members
- ☐ Others

MUSTER

MUSTER

4. Questions about medical research projects [Fortsetzung]

4.11

4.12 What are your motives agreeing to participate in medical research projects?
Multiple choice possible

- ☐ Hope for personal benefit
- ☐ Last hope/desperation
- ☐ Gain new knowledge to help future generations
- ☐ Gain new knowledge to help other patients
- ☐ Trust in doctors/researchers/scientists
- ☐ Others

4.13

4.14 What are your motives not agreeing to participate in medical research projects?
Multiple choice possible

- ☐ Incalculable risk
- ☐ Too much burden on your own
- ☐ Too much time required
- ☐ No direct benefit
- ☐ Own illness to advanced
- ☐ No sense recognized in the study
- ☐ Incomprehensible methods
- ☐ Others

4.15

MUSTER
