# Supplementary material for: Attitudes of legal guardians and legally supervised persons with and without previous research experience towards participation in research projects: A quantitative cross-sectional study
Source: PLoS One. 2021 Sep 15;16(9):e0256689. doi: 10.1371/journal.pone.0256689 (PMC8443074; doi:10.1371/journal.pone.0256689)
Supplement: S4 File — (PDF) [file pone.0256689.s004.pdf]

EvaSys	Fragebogen betreute Personen [Copy]	Electric Paper

Universitätsmedizin Greifswald

Institut für Community Medicine, Abteilung Betreuerstudie 2019

Bitte so markieren: ☐ ☒ ☐ ☐ ☐
Korrektur: ☐ ■ ☐ ☒ ☐

1. Aufklärung

Sehr geehrte Teilnehmerin, Sehr geehrter Teilnehmer,

vielen Dank, dass Sie sich die Zeit nehmen den nachfolgenden Fragebogen zu beantworten.

Mit Ihrer Hilfe möchte wir, Wissenschaftler der Universitätsmedizin Greifswald, untersuchen, welche Gründe aus Ihrer Sicht für bzw. gegen eine Teilnahme an wissenschaftlichen Studien im Bereich Gesundheit und Versorgung sprechen.

In Zukunft wird es immer wichtiger, dass auch Menschen mit einer gesetzlichen Betreuung an Forschungsprojekte teilnehmen können. Das ist wichtig, weil auch Menschen mit einer Betreuung an Fortschritten in der medizinischen Versorgung teilhaben können müssen.

In diesem Fragebogen werden Ihnen zunächst einige Fragen zu Ihrer Betreuungssituation gestellt. Danach möchten wir etwas über Ihre Gründe zur Zustimmung oder Ablehnung einer Teilnahme an wissenschaftlichen Projekten erfahren.

Es gibt bei der Beantwortung der Fragen kein „richtig“ oder „falsch“, nur Ihre persönliche Meinung zählt.
Die Beantwortung der nachfolgenden Fragen wird etwa 8 Minuten in Anspruch nehmen.

Ihre Daten werden von uns streng vertraulich behandelt. Alle Daten unterliegen dem Datenschutz und werden ausschließlich anonymisiert verarbeitet und nicht an Dritte weitergegeben. Ihre Daten dienen ausschließlich dem wissenschaftlichen Zweck dieser Befragung und werden anschließend nicht weiterverwendet. Die Teilnahme an der Befragung ist freiwillig. Sie dürfen zu jedem Zeitpunkt, ohne Angaben von Gründen, die Teilnahme beenden.

Sollten Sie Fragen zu dieser Befragung haben, kontaktieren Sie uns gerne per E-Mail (betreuerstudie@uni-greifswald.de).

1.1
☐ Ich habe den obigen Text zur Kenntnis genommen und bin mit der Teilnahme an der Studie einverstanden.

2. Allgemeine Fragen

2.1 Bitte geben Sie Ihr Alter an
☐ unter 30 ☐ 30-40 ☐ 41-50
☐ 51-60 ☐ 61-70 ☐ 71-80
☐ 81-90 ☐ über 90

2.2 Bitte geben Sie Ihr Geschlecht an
☐ weiblich ☐ männlich ☐ divers

2.3 Wie ist Ihr Familienstand?
☐ ledig ☐ verheiratet ☐ dauernd getrennt lebend
☐ geschieden ☐ verwitwet

2.4 Was sind/ waren Sie von Beruf?

[ ]

2.5 Wie ist Ihre aktuelle Wohnsituation?
☐ Eigenständige Haushaltsführung ☐ Haushaltsführung zusammen mit weiteren Personen ☐ Eigener Haushalt, durch mobilen Pflegedienst betreut
☐ In betreutem Wohnheim wohnend ☐ Sonstige

2.6

[ ]

3. Betreuungssituation

3.1 Seit wann werden Sie gesetzlich betreut? (in Jahren)
☐ unter 1 ☐ 1-3 ☐ 4-6
☐ 7-9 ☐ 10 oder länger

3.2 Seit wann werden Sie von Ihrem aktuellen Betreuer betreut? (in Jahren)
☐ unter 1 ☐ 1-3 ☐ 4-6
☐ 7-9 ☐ 10 oder länger

3.3 Was ist der Grund Ihrer Betreuung?

[ ]

3. Betreuungssituation [Fortsetzung]

3.4 Für welchen Bereich besteht die Betreuung?
Mehrfachnennung möglich

- ☐ Vermögensregelung
- ☐ Gesundheitsfürsorge
- ☐ Heimangelegenheiten
- ☐ Wohnungsangelegenheiten
- ☐ Behördenangelegenheiten
- ☐ Sonstige
- ☐ weiß nicht

3.5

3.6 Wie würden Sie das Verhältnis zu Ihrem Betreuer beschreiben?

- ☐ emotional
- ☐ freundschaftlich
- ☐ sachlich
- ☐ sonstiges

3.7

4. Fragen zur wissenschaftlichen Forschung

4.1 Wurden Sie in der Vergangenheit schon einmal aufgefordert an einem wissenschaftlichen Projekt teilzunehmen?

- ☐ ja
- ☐ nein

4.2 Um welche Art von Studie handelte es sich?

- ☐ Arzneimittelstudie
- ☐ Computertestverfahren (bspw. Test zur Feststellung des IQ oder Reaktionsvermögens)
- ☐ Bildgebende Studie (bspw. MRT-Untersuchung zur Darstellung veränderter Hirnstrukturen bei Demenzpatienten)
- ☐ Untersuchung genetischer Marker (wichtig bei Krankheiten mit erblicher Komponente und zur Erkennung verschiedener Erkrankungen)
- ☐ Interview
- ☐ Blutentnahme zu Zwecken medizinischer Forschung
- ☐ Telemedizinische Studie (bspw. Führung von Arzt-Patienten-Gesprächen via Video-Telefonat oder Kontaktaufnahme via SMS)
- ☐ Sonstige

4.3

4.4 Haben Sie der Teilnahme zugestimmt?

- ☐ ja
- ☐ nein

4.5 Wer hat die Entscheidung getroffen?

- ☐ Sie selbst
- ☐ Der Betreuer
- ☐ Gemeinsam
- ☐ Sie unter Einbeziehung von Familienangehörigen
- ☐ Sonstige

4.6

4.7 Wären Sie grundsätzlich bereit an einem wissenschaftlichen Forschungsprojekt teilzunehmen?

- ☐ ja
- ☐ nein

4.8 Bei welcher Art von Studie könnten Sie sich vorstellen teilzunehmen?
Mehrfachnennung möglich

- ☐ Arzneimittelstudie
- ☐ Computertestverfahren (bspw. Test zur Feststellung des IQ oder Reaktionsvermögens)
- ☐ Bildgebende Studie (bspw. MRT-Untersuchung zur Darstellung veränderter Hirnstrukturen bei Demenzpatienten)
- ☐ Untersuchung genetischer Marker (wichtig bei Krankheiten mit erblicher Komponente und zur Erkennung verschiedener Erkrankungen)
- ☐ Interview
- ☐ Blutentnahme zu Zwecken medizinischer Forschung
- ☐ Telemedizinische Studie (bspw. Führung von Arzt-Patienten-Gesprächen via Video-Telefonat oder Kontaktaufnahme via SMS)
- ☐ Sonstige

4.9

4. Fragen zur wissenschaftlichen Forschung [Fortsetzung]

4.10 Wer würde die Entscheidung voraussichtlich treffen?

- ☐ Sie selbst
- ☐ Der Betreuer
- ☐ Gemeinsam
- ☐ Sie unter Einbeziehung von Familienangehörigen
- ☐ Sonstige

4.11

[ ]

4.12 Was war bzw. was würden Sie als Grund für Ihre Zustimmung sehen?
Mehrfachnennung möglich

- ☐ Hoffen auf persönlichen Nutzen
- ☐ Letzte Hoffnung/ Verzweiflung
- ☐ Gewinn neuer Erkenntnisse um Nachkommen in Zukunft helfen zu können
- ☐ Gewinn neuer Erkenntnisse um anderen Leidenden helfen zu können (Altruismus)
- ☐ Vertrauen in ärztliche Tätigkeit
- ☐ Sonstige

4.13

[ ]

4.14 Was war bzw. was würden Sie als Grund für Ihre Ablehnung sehen?
Mehrfachnennung möglich

- ☐ Risiko nicht abschätzbar
- ☐ Zu große Belastung Sie selbst
- ☐ Zu großer zeitlicher Aufwand
- ☐ Kein direkter Nutzen für Sie selbst
- ☐ Krankheit zu weit fortgeschritten
- ☐ keine Sinnhaftigkeit in der Studie erkannt
- ☐ Methodik unverständlich
- ☐ Sonstige

4.15

[ ]
